# Uptake of Immediate Postpartum LARCs and Associated Factors among Mothers Who Gave Birth at Hawassa University Comprehensive Specialized Hospital, Hawassa, Ethiopia

**DOI:** 10.1155/2022/1422094

**Published:** 2022-07-07

**Authors:** Mequanent Tariku, Biruk Legesse, Temesgen Tantu, Bereket Duko

**Affiliations:** ^1^Department of Obstetrics and Gynecology, Debre Tabor University, College of Medicine and Health Sciences, Ethiopia; ^2^Department of Obstetrics and Gynecology, Assela University, College of Medicine and Health Sciences, Ethiopia; ^3^Department of Obstetrics and Gynecology, Wolkite University, College of Medicine and Health Sciences, Ethiopia; ^4^Curtin School of Population Health, Curtin University, Western Australia, Australia

## Abstract

**Background:**

Postpartum family planning is an effective strategy for reducing maternal and childhood morbidity and mortality by preventing unintended pregnancy and short interpregnancy intervals. Despite the paramount advantages of long-acting reversible contraceptives (LARC), their uptake remains low in Ethiopia. Therefore, the aim of this study was to assess the uptake of immediate postpartum LARC methods and its associated factors among women who gave birth in Hawassa University Comprehensive Specialized Hospital, Hawassa city, Southern Ethiopia.

**Methods:**

An institution-based cross-sectional study was conducted among 418 eligible mothers who were in the immediate postpartum period. Data were collected using a pretested structured questionnaire before their discharge from the hospital and analyzed by using SPSS version 20. The statistical significance was declared at *P* value less than 0.05.

**Results:**

The uptake LARCs among immediate postpartum mothers was 25.4%. The most commonly reported reasons for not using LARC were preference to start contraception after six weeks of delivery (43.3%) and the need to use other methods of contraception (26%). Having unplanned birth (AOR: 1.97; 95% CI: 1.04-3.71) and receiving family planning counselling on LARCs during the postpartum period (AOR: 21.1; 95% CI: 6.49-68.66) were factors significantly associated with immediate postpartum LARC use.

**Conclusion:**

Low utilization of immediate postpartum LARC uptake was found in the current study setting. There was increased utilization of immediate postpartum LARC among mothers who received family planning counselling during the postpartum period. Therefore, strengthening family planning counselling during the immediate postpartum period is crucial to enhance postpartum LARC use.

## 1. Introduction

Family planning is a highly effective public health intervention with the potential to reduce both maternal and newborn morbidity and mortality, yet the unmet need for modern contraceptives remained high. Postpartum family planning is the prevention of unintended and closely spaced pregnancies during the first twelve months following childbirth [1, 2]. Long-acting reversible contraception (LARC) methods, which include intrauterine devices (IUDs) and contraceptive implants, are the most effective reversible methods available to women, with failure rates of less than 1% [1–4]. According to the existing studies, non-LARC users were more than 22 times more likely to experience unintended pregnancy compared to their LARC counterparts as well; they do not require ongoing effort from the woman for long-term and effective use, and return of fertility is rapid after removal [1–6].

Postpartum women are among those with the greatest unmet need for family planning. Analysis of the Demographic Health Survey (DHS) of 27 countries including Ethiopia revealed that many postpartum women have a high unmet need for family planning (FP) during the first year after childbirth and they did not receive the service (3, 4, 9). However, the World Health Organization (WHO) recommends that waiting 24 months after birth before attempting the next pregnancy [7] can avert an estimated 30% of maternal deaths, and 10% of infant deaths [8] could greatly reduce maternal mortality and morbidity by 75%, unwanted pregnancies by two-thirds, and risks of abortion by 74% [1, 9–11].

Analysis of DHS data from 21 low- and middle-income countries (LMIC) revealed that only 31% of all postpartum women used family planning [12–14]. And the majority of these women used short-acting methods (51–96% of postpartum contraceptive users). According to this survey and multiple local studies, among women in their first two years postpartum in Ethiopia, only 20% were using methods of family planning, and the mix method was dominated by short-acting methods; few (12%) were using LARC and permanent methods, while a majority (85%) were using short-acting methods [12–15].

Based on a few available studies, several factors that influence immediate postpartum LARC uptake have been reported. Having family planning counselling during ANC and the immediate postpartum period, level of education, partner support, and prior knowledge on LARC are among factors that increase the rate of utilization LARC while fear of side effects, use of other methods, desire to have more children, and opposition from a partner are some of the reasons mentioned to decline LARC use in the immediate postpartum period [13, 15–22].

In Ethiopia, information and UpToDate evidence on the utilization of LARC during the immediate postpartum period are scarce. Therefore, the current study examined the uptake and factors that facilitate or impede the utilization of LARC among women in the immediate postpartum period at Hawassa University Comprehensive Specialized Hospital, Ethiopia.

## 2. Methods and Materials

### 2.1. Study Area, Design, and Populations

Institution based cross sectional study was conducted at Hawassa University Comprehensive Specialized Hospital, Hawassa City, Ethiopia, from January 1, 2019 to March 30, 2019. Hawassa City is situated at 273 KM south east to the capital of Ethiopia. All mothers who gave birth at Hawassa University Comprehensive Specialized Hospital (HUCSH) during the study period and in the immediate postpartum period were included in the current study.

### 2.2. Sample Size Determination and Sampling Technique

The required sample size for this study was calculated using single population proportion formula and with an assumption of 95% confidence interval, and 5% margin of error, a proportion of 45% from study done at Saint Paul Millennium Medical College, Ethiopia [18] on postpartum uptake of LARC and tubal ligation. Adding 10% contingency to account for non-response rate yielded a final sample size of 418. Systemic random sampling method was used. The total number of mothers who gave birth in HUCSH three months prior to the study period was 1244 (eligible population), dividing this with 418 (required sample size), yielded 3. The first study participant was selected by lottery method, and then every 3rd study participant was included into the study until the study period ends.

### 2.3. Inclusion and Exclusion Criteria

#### 2.3.1. Inclusion Criteria

All eligible and consenting mothers in the immediate postpartum period after giving birth in HUCSH during the study period were included in the study.

#### 2.3.2. Exclusion Criteria

Mothers in the immediate postpartum period who did not fulfill the WHO medical eligibility criteria for immediate postpartum LARC use [7], came after giving birth at other health institutions or home, others who had tubal ligation during cesarean delivery, seriously ill, and who were not able to communicate during data collection period were excluded.

### 2.4. Data Collection Instruments

Data were collected by four medical interns and two year one residents by interviewing postpartum mothers before discharge from the postnatal unit or wards using a pretested structured questionnaire which was prepared after review of available literatures. The medical interns and year one residents were trained for one day on objective of the study and method of data collection. To assure the quality of data, data collectors were trained before the actual study period. Pretesting of the questionnaire was conducted on twenty-one postpartum women at Adare Hospital before the study period, and appropriate modifications were made. In addition, regular checkup for completeness, accuracy, and consistency of the data was made on daily basis by the principal investigator.

### 2.5. Data Processing and Analysis

The data were checked for completeness, cleaned, and coded for entry and analysis by principal investigator. Data were analyzed using Statistical Package of Social Sciences (SPSS) version 20. Descriptive statistics (frequencies and percentages) were used to explain the study participant in relation to study variables. Bivariate and multivariate analyses were used to determine the presence of statistically significant associations between variables. The strength of the association was presented by odds ratio and 95% confidence interval. A *P* value set at 0.05 on multivariate analyses was considered as statistically significant.

#### 2.5.1. Operation Definitions

Contraceptive prevalence rate is the percentage of currently married women aged 15-49 years who are using any method of family planning.

Awareness about LARC is a woman in immediate postpartum period that know and/or used at least one of the LARC methods, i.e., Implanon, Jadelle, and/or IUCD.

Immediate post-partum period refers to the first 48 hours after childbirth.

Unmet need for family planning is the percentage of women who want to stop or delay childbearing but who are not using any method of contraception to prevent pregnancy.

Unmet need for modern family planning is the percentage of women who want to avoid a pregnancy but are currently using no method or a traditional contraceptive method.

## 3. Results

### 3.1. Sociodemographic Characteristics

More than half (54.1%) of the respondents were in the age range of 25 to 34 years. Additionally, the majority of participants were married (99.3%), and more than two-thirds (66.7%) were urban residents (Table 1).

### 3.2. Obstetric Characteristics of Respondents

Of the total respondents, majority (41.9%) of the mothers were primiparas, and nearly one-sixth participants had four and above live children. Twenty-three percent of the mothers got pregnant before two years of their previous delivery. Additionally, about three-fourths (74.6%) of the participants have planned to have more children soon (Table 2).

### 3.3. Uptake of Immediate Postpartum LARC

The prevalence of postpartum LARCs (LARC) was 25.4% (95% confidence interval: 21.1% to 29.5%). Implants were the most commonly used methods (76.4%). Majority (82.8%) of the respondents had information about LARC, predominantly from health workers (41.6%) followed by health extension workers (20.5%). On the other hand, only 79 (18.9%) mothers used these methods previously. More than one-fourth (27.8%) of the participants were not counseled about LARC methods during postnatal period (Table 3).

LARC Long Acting Reversible Contraceptives; IUCD Intra-uterine contraceptive device.

**Figure 1:**
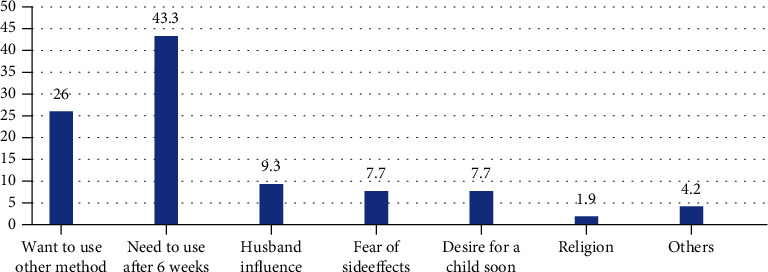
Reasons for not to use postpartum LARCs in HUCSH, 2019.

The most common reason for not using postpartum LARC methods was mothers' preference to use family planning methods after 6 weeks of delivery. Twenty-six percent of partcipants were not using LARC as they wanted to use other family planning methods. Husband education (9.3%), fear of side effects (7.7%), and religion (1.9%) were other reported reasons by the respondents (Figure 1).

### 3.4. Factors Associated with Uptake of Postpartum LARC

All candidate variables in the chi-square test were computed in binary logistic regression. In the binary logistic regression analysis, maternal age, ethnicity, maternal occupation, intention of index birth, number of ANC visits, information about LARC, previous use of LARC, counseling about family planning methods during ANC visits, counseling about LARC at ANC visit, and postnatal period were variables whose *P* value was ≤0.25 (Table 4).

In the multivariable logistic regression, only planned birth and counseling about LARC methods during postnatal period remained independent predictors of LARC uptake. Mothers who had unplanned birth were 1.97 more likely to use postpartum LARC methods (AOR: 1.97; 95: CI 1.04-3.71). Similarly, mothers who got counseling about LARC methods during the postnatal period were 21 times (AOR: 21.1; 95% CI 6.49-68.66) more likely to use these methods, than their counterparts who were not counseled after delivery (Table 4).

## 4. Discussion

It was found that 25.4 percent of mothers started using immediate postpartum LARC. Unplanned birth and counseling about LARC during postnatal period showed statistically significant association with uptake of immediate postpartum LARC. This finding was in line with evidences from studies conducted in Addis Ababa (29.2%) [8], Uganda (28%) [23], Pakistan (27%) [14], Texas (23%) [24], and Adaba (30.3%) [25] though the study populations were different. However, this finding is much lower than findings of studies conducted in Kenya [17] and Jimma [26], which reported the prevalence of LARC among immediate postpartum mothers as 57%, 45%, and 53%, respectively. The inconsistencies may be explained by the differences in residence of study population and perinatal service utilization. For instance, the proportion of mothers who received postpartum family planning counseling was lower in this study (72%) than the study done in Kenya (100%). The most commonly used methods by this study participants were implants (76.4%) followed by IUCD (23.6%). A comparable finding was reported in Durame town where 80% and 20% of postpartum women used implants and IUCD, respectively [13].

Majority (74.6%) of postpartum women did not start using LARC. The reasons for this non-use of postpartum LARCs include need to use after 6 weeks, want to use other methods, and husband influence, which accounted for 43.3%, 26%, and 9.3%, respectively. This finding is in line with a study conducted in Saint Paul's Hospital Millennium Medical College of Ethiopia, where partner opposition and preference of other methods were the commonest mentioned reasons for non-use of LARC [27]. Similarly, in Jimma, 26% of women reported preference of other method as a major reason for non-use of LARC [28].

The present study showed that mothers with unplanned births were 2 times more likely to use immediate postpartum LARCs compared to those mothers with planned births. This is in line with a study done in Kenya which revealed that women with last unintended pregnancies were more likely to use modern contraceptive methods than women whose last pregnancies were intended [29]. This could be explained by the fact that mothers who experienced unplanned pregnancy may not want to repeat similar episode, give great emphasis for pregnancy prevention, and require long-acting reversible contraception. Immediate postpartum period is an attractive coincidence, particularly for those at high risk of short inter pregnancy intervals, due to the reason that most women have low postnatal care visits [30]. Further, it eliminates possible barriers, including the need for additional visits that hinder utilization of the methods.

Immediate postpartum counseling about LARC was the other most significantly associated factor for uptake of postpartum LARC. Compared to mothers who were not counseled about LARC during immediate postpartum period, mothers who received the message were 21 times more likely to start utilizing these contraceptive methods. This finding is consistent with similar studies [28, 31]. Similar other study found that mothers who received counseling service on LARC during postpartum period were four times more likely to use the method compared to those never received the counseling service [18]. Similarly, another study reported that immediate postpartum counseling was a significant predictive factor for LARC use [30]. Moreover, in a North American study, those women with postpartum counseling were reported higher utilization rate of LARC [32].

The current study also showed that antenatal counseling about LARC did not have significant association with utilization of LARC during immediate postpartum period. A similar finding was reported by a study done in Uganda [33] and Addis Ababa [18] . This may be due to women's priority concern for health of their fetus and complications during pregnancy. In contrary to this, a study done in Mexico reported that, compared to those women who did not receive prenatal counseling, those who received counseling were two times more likely to use postpartum modern contraceptive methods [21].

### 4.1. Strength of the Study

The study is the first to assess uptake of immediate postpartum LARC in the study area.

### 4.2. Limitations of the Study

The cross-sectional nature of the study does not allow the study to establish causal relationship between different independent variables and outcome variable. Furthermore, the study was conducted in tertiary center; therefore, the findings of the study might not be representative of the general population. The study did not address all health system–related factors that affect postpartum utilization LARCs.

## 5. Conclusion and Recommendations

Low utilization of immediate postpartum LARC uptake was found in this study. There was increased utilization of immediate postpartum LARC among mothers who received family planning counseling during postpartum period. Therefore, strengthening family planning counseling during immediate postpartum period is crucial to enhance postpartum LARC use. Further study is recommended on health service provider related factors associated with uptake of immediate postpartum LARCs.

## Figures and Tables

**Table 1:** Sociodemographic characteristics of mothers who gave birth at HUCSH, Hawassa, Ethiopia, April 2019.

Variables	Frequency (*n*)	Percent (%)
Mothers age (*n* = 418)		
15-24	148	35.4
25-34	226	54.1
35-44	44	10.5
Religion (*n* = 418)		
Christian	306	73.2
Muslim	109	26.1
Others	3	0.7
Ethnicity (*n* = 418)		
Oromo	141	33.7
Amhara	67	16.0
Sidama	131	31.3
Wolayita	35	8.4
Others	44	10.5
Place of residence (*n* = 418)		
Rural	136	32.5
Urban	282	67.5
Marital status (*n* = 418)		
Married	415	99.3
Single/divorced	3	0.7
Mothers' occupation (*n* = 418)		
Housewives	223	53.3
Merchant	66	15.8
Farmer	15	3.6
Student	23	5.5
Government employee	82	19.6
Daily laborer	5	1.2
Others	4	1.0
Husband occupation (*n* = 416)		
Merchant	139	33.4
Farmer	74	17.8
Student	7	1.7
Government employee	145	34.9
Daily laborer	29	7.0
Others	22	5.3
Mothers' education (*n* = 418)		
Never attended school	44	10.5
Primary (1–8)	161	38.5
Secondary (9, 10)	103	24.6
Above secondary	110	26.3
Husband education (*n* = 416)		
Never attended school	12	2.9
Primary school (1–8)	123	29.6
Secondary school (9, 10)	89	21.4
Above secondary school	192	46.2

**Table 2:** Obstetric profile of respondents in HUCSH, Ethiopia, April 2019.

Variables	Frequency (*n*)	Percent (%)
Parity (*n* = 418)		
One	175	41.9
Two	107	25.6
Three	59	14.1
Four and above	77	18.4
Number of children (*n* = 418)		
One	194	46.4
Two	94	22.5
Three	61	14.6
Four and above	65	15.6
None	4	1.0
Birth to pregnancy interval (*n* = 243)		
< two years	56	23.0
≥ two years	187	77.0
Planned pregnancy (current) (*n* = 418)		
Yes	338	80.9
No	80	19.1
Plan to have more children (*n* = 418)		
Yes	312	74.6
No	55	13.2
Undecided	51	12.2
Future plan of birth (*n* = 312)		
Before two years	13	4.2
After two years	240	76.9
Undecided	59	18.9
ANC follow-up (*n* = 418)^†^		
Yes	401	95.9
No	17	4.1
Number of ANC visits (*n* = 401)		
One	4	1.0
Two	44	11.0
Three	147	36.7
Four and above	206	51.4
Mode of delivery (*n* = 418)		
SVD	267	63.9
Caesarean section	146	34.9
Forceps/vacuum delivery	5	1.2
Birth outcome on discharge (*n* = 418)		
Alive	390	93.3
Dead	28	6.7

^†^At least one; SVD: spontaneous vertex/vaginal delivery.

**Table 3:** Awareness and utilization of LARC methods among women who gave birth in HUCSH, Ethiopia, April 2019.

Variables	Frequency (*n*)	Percent (%)
Ever heard about LARCs (LARC) (*n* = 418)		
Yes	346	82.8
No	72	17.2
Source of information about LARC (*n* = 346)		
Health worker	144	41.6
Health extension worker	71	20.5
Friends	57	16.5
TV/radio	69	19.9
Others	5	1.4
Have you ever used LARC? (*n* = 418)		
Yes	79	18.9
No	339	81.1
Previously used methods of LARC (*n* = 79)		
Implants	56	70.9
IUCD	22	27.8
Both methods	1	1.3
Counseled about family planning during ANC visits (*n* = 401)		
Yes	212	52.9
No	189	47.1
Counseled about LARC during ANC visits (*n* = 401)		
Yes	157	39.2
No	244	60.8
Counseled about LARC during postnatal period (*n* = 418)	302	72.2
Yes		
No	116	27.8
Used immediate postpartum LARC (*n* = 418)		
Yes	106	25.4
No	312	74.6
Type of LARC method used (*n* = 106)		
IUCD	25	23.6
Implants	81	76.4

**Table 4:** Factors associated with uptake of postpartum LARC methods in HUCSH, Ethiopia, April 2019.

Variables	Uptake of LARC *n* (%)	Crude odds ratio (95% CI)	Adjusted odds ratio (95% CI)
Mothers age			
15-24	36 (34.0)	1	1
25-34	53 (50.0)	0.95 (0.587, 1.54)	0.93 (0.54, 1.61)
35-44	17 (16.0)	1.95 (0.96, 3.99)	1.46 (0.64, 3.30)
Ethnicity			
Oromo	29 (27.4)	1.00 (0.43, 2.32)	1.02 (0.41, 2.54)
Amara	23 (21.7)	2.03 (0.83, 4.94)	2.33 (0.89, 6.08)
Sidama	36 (34.0)	1.47 (0.64, 3.36)	1.31 (0.53, 3.18)
Wolayita	9 (8.5)	1.34 (0.46, 3.86)	1.10 (0.35, 3.44)
Others	9 (8.5)	1	1
Mothers occupation			
Unemployed	58 (54.7)	1	1
Employed	48 (45.3)	1.41 (0.90, 2.21)	1.29 (0.78, 2.14)
Planned birth			
Yes	82 (77.4)	1	1
No	24 (22.6)	0.74 (0.43, 1.28)	**1.97 (1.04, 3.71)** ^∗^
Number of ANC visits			
1-3	55 (52.9)	1	1
4 and above	49 (47.1)	0.79 (0.50, 1.24)	0.71 (0.43, 1.16)
Heard about LARC			
Yes	93 (87.7)	1.66 (0.87, 3.18)	1.03 (0.46, 2.31)
No	13 (12.3)	1	1
Previous use of LARC			
Yes	24 (22.6)	1.36 (0.79, 2.34)	1.19 (0.65, 2.19)
No	82 (77.4)	1	1
Counseled about FP methods during ANC visits			
Yes	58 (55.8)	1.17 (0.74, 1.83)	0.98 (0.59, 1.60)
No	46 (44.2)	1	1
Counseled about LARC during ANC			
Yes	45 (43.3)	1.26 (0.80, 1.98)	1.06 (0.48, 2.32)
No	59 (56.7)	1	1
Counseled about LARC during postnatal period			
Yes	103 (97.2)	19.49 (6.04, 62.87)	**21.1 (6.49, 68.66)** ^∗∗^
No	3 (2.8)	1	1

CI: confidence interval, ∗*P* value < 0.05; ∗∗*P* value less than 0.001; Hosmer and Lemeshow goodness-of-fit =0.41.

## Data Availability

All data were included in the manuscript.

## Abbreviations

HUCSH: Hawassa University Comprehensive Specialized Hospital
IUCD: Intrauterine contraceptive device
LMIC: Low- and middle-income countries
ANC: Antenatal care.
